# Local Vibratory Stimulation for Temporomandibular Disorder Myofascial Pain Treatment: A Randomised, Double-Blind, Placebo-Controlled Preliminary Study

**DOI:** 10.1155/2020/6705307

**Published:** 2020-12-05

**Authors:** Emanuela Serritella, Giordano Scialanca, Paola Di Giacomo, Carlo Di Paolo

**Affiliations:** Clinical Gnathology Unit, Department of Oral and Maxillofacial Sciences, “Sapienza” University of Rome, Rome, Italy

## Abstract

Several methods are currently used to manage pain related to temporomandibular disorder (TMD). Vibratory stimulation is applied as a pain treatment for several musculoskeletal disorders, but it has not yet been studied in-depth for TMD symptoms. The aim of this study is to analyse the effectiveness of at-home local vibration therapy (LVT) for the management of TMDs-related myofascial pain. *Methods*. Fifty-four TMD patients (43 F, 11 M) with an average age of 40.7 (age range: 29–54 yr.) were randomly subdivided into two groups. The study group (AG) received 1 week of at-home LVT treatment with the NOVAFON Pro Sk2/2 : 50/100 Hz, bilaterally applied to the pain area for 16 minutes daily. The placebo group (IG) followed the same protocol using inactive devices. Temporomandibular joint pain (TMJ), muscular pain (MM), and headache (HA) were assessed. Pain was evaluated using the visual analogue scale (VAS) before (T0) and after therapy (T1). Statistical analysis and Student's *t*-tests were applied (statistical significance for *P* < 0.05). *Results*. AG patients reported decreased average values for all types of pain considered between T0 and T1, with a statistically significant difference for TMJ pain (*P* < 0.05), MM pain, and HA (*P* < 0.001). IG patients reported a no statistically significant decrease in the average values of MM pain and an increase in the average values of TMJ pain and HA. *Conclusion*. The study supports the use of local vibration therapy in the control of TMD-related TMJ pain, local muscular pain, and headache.

## 1. Introduction

Temporomandibular disorders (TMD) comprise a large number of pathologies related to the masticatory muscles and/or temporomandibular joint (TMJ) and constitute a part of the musculoskeletal disorder group [1]. Current indications for treatment of these conditions follow a conservative approach that includes information, reassurance, control of functional excesses, physiotherapy rehabilitation, application of physical therapies, administration of drugs, and intraoral devices [1–3]. Like other musculoskeletal disorders, TMD has been treated in recent years with various physical therapy methods, in cases with different types of TMD pain (pain-related, intraarticular, degenerative groups). TENS (transcutaneous nerve electrical stimulation) and low-level laser therapy (LLLT) are among the most utilized treatment procedures [4–6].

One of the most recently proposed physical therapy treatments is local vibration therapy. Local vibration therapy produces vibrations that reach up to 6 centimetres of tissue depth; it is used to regulate muscle tone, relieve localized pain, and stimulate an increase in blood and lymphatic circulation [7–9]. This therapy is most frequently applied in the treatment of chronic pathologies affecting the muscles, tendons, and joints. Several studies evaluating the impact of local vibration therapy on skeletal muscles and joints have highlighted its effectiveness for increasing joint mobility and decreasing pain [10, 11], but analysis of its potential for the temporomandibular region is still lacking. Only two studies have addressed the application of this therapy to TMD and both demonstrate its effectiveness for muscle pain relief [12, 13].

The aim of this study is to evaluate the effectiveness and efficiency of local vibration therapy in the treatment of craniomandibular pain by comparing the application of an active vibratory device with the application of an inactive placebo device on two samples of dysfunctional patients.

## 2. Materials and Methods

A randomized, double-blind, placebo-controlled clinical study was conducted at the Clinical Gnathology Unit of the Department of Oral and Maxillofacial Sciences at the “Sapienza” University of Rome. The study was approved by the Institutional Ethics Committee (N. 93/2017-0001385); all patients signed an informed consent document before participating in the study.

### 2.1. Participants

**Figure 1:**
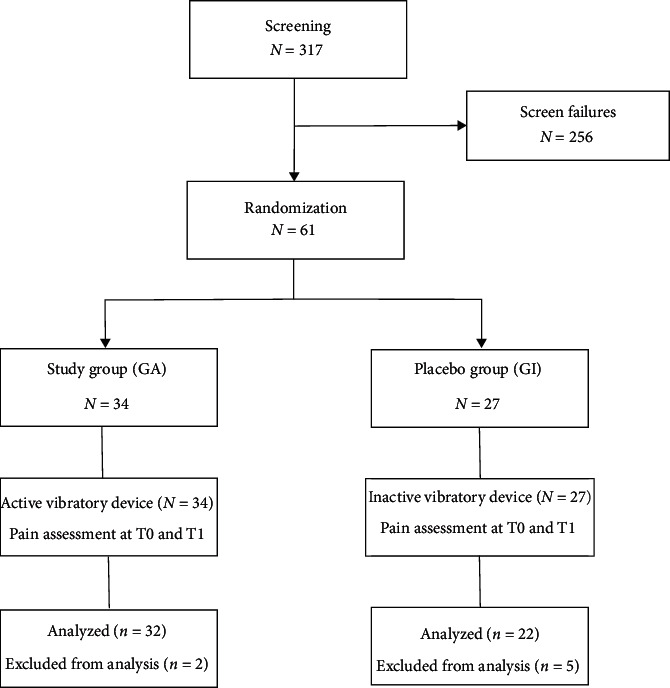
CONSORT flow diagram of patient enrollment and interventions.

The patient enrollment process followed the CONSORT (Consolidated Standards of Reporting Trials) criteria (Figure 1).

During the period of February 2018–July 2019, 317 subjects under observation in our department were assessed for eligibility. All patients were screened for temporomandibular disorders (TMD) by specialists in the field using the DC/TMD diagnostic criteria [14]. Criteria for inclusion in the study were as follows: (1) diagnosis of chronic local myalgia (ICD-9 729.1) with average reported pain greater than or equal to 3 on the numeric verbal scale (NVS); (2) availability to participate in the study; and (3) current residence in Rome or the surrounding province. Patients meeting the following exclusion criteria were excluded from the study: (1) diagnosed with widespread pain; (2) diagnosis of joint disorders (ICD-9 524.63; ICD-9 715.18; and ICD-9 830.0); and (3) receiving ongoing gnathological treatment. Following manufacturer indications for the therapeutic device, additional exclusion criteria were also applied: (1) presence of open wounds/eczema on the skin or the skin membranes involved in the treatment; (2) diagnosis of arteriosclerosis, thrombosis, cardiac arrhythmias, or use of a pacemaker; (3) diagnosis of epilepsy; (4) use of brain stimulators or presence of metal implants; (5) presence of tumour lesions; and (6) pregnant women.

256 patients were excluded according to these criteria. The resulting study sample consisted of 61 patients, 16 male (26.2%) and 45 female (73.8%), with an average age of 38.39 years (range 29–54 years).

### 2.2. Interventions

The study involved the administration to all patients of a local vibration device (NOVAFON Pro (Sk2)) for professional/home mixed use. Patients were treated with both active, functioning devices and placebo devices identical to the functioning ones but therapeutically inactive. The therapeutic protocol involved 7 applications of vibration therapy: the first and last applications were performed by a specially trained operator (G.S.) at the clinical gnathology department; the remaining 5 were carried out at home by the patient.

A single operator (G.S.), blinded to the diagnosis and symptoms of patients, carried out the distribution of the devices and provided patient instruction on correct methods of use; all patients were given the same instructions for home use following the indications provided by the manufacturer. Patients used the active or placebo device for 5 days for 16 minutes a day.

The symptoms evaluated for all patients were joint pain, muscular pain (masticatory muscle pain), and headache (attributed to TMD). Each type of pain was measured at the following times:

- T0 : before treatment
- T1 : after the last application (7 days after T0)

The 0–100 visual analogue scale (VAS) was used to measure pain self-assessment, with 0 indicating “no pain” and 100 “the worst imaginable pain.”

**Figure 2:**
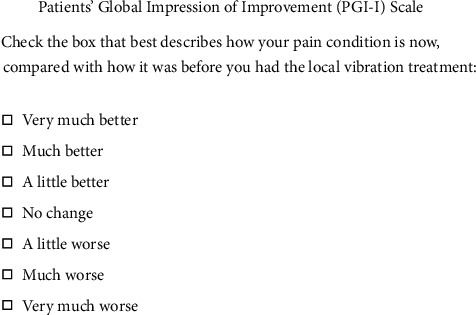
PGI-I Scale used to evaluate patients' impression of improvement.

At the end of treatment (T1), all patients were given a questionnaire regarding their impression of the treatment's effectiveness: Patients' Global Impression of Improvement (PGI-I) Scale (Figure 2).

In order to perform a comparative data analysis of the active and inactive devices, all participants were subsequently divided into two groups: a study group (AG) that received active devices and a placebo group (IG) that received inactive devices.

The primary outcome of the study was to evaluate the change in perceived pain levels after one week of local vibration therapy in the group that received active devices (AG) and in the group that received placebo devices (IG).

### 2.3. Local Vibration Device and Application Procedure

The device used was the NOVAFON Pro Sk2/2 (NOVAFON GmbH, Weinstadt).

**Figure 3:**
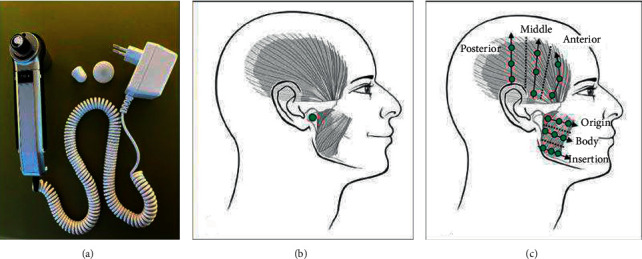
NOVAFON Pro Sk2/2 (a); application points used at the (b) temporomandibular joint; (c) masseter muscle and temporalis muscle.

This direct current electromedical device consists of a switch with two levels to adjust the intensity of the vibration produced (50/100 Hz); a handpiece to modify the power of the vibration; spherical and disc-shaped extra oral heads (means of stimulating the skin and mucous membranes); and an extension clamp (Figure 3(a)).

Two different application modalities were applied on both sides of the face to the masseter (deep and superficial) and temporal (anterior, middle, and posterior) muscles and to the TMJ [1], for a total of 16 minutes per day (Figures 3(b) and 3(c)):

1. Use of the disc head on button 2 (50 Hz) for 4 minutes/side. The device was used with moderate pressure and rotational movements along the masseter and temporal muscles. The disc surface allows for greater dispersion of vibratory stimulation, with the aim of relaxing the musculature.
2. Use of the spherical head on button 1 (100 Hz) for 4 minutes/side. The device was used with moderate pressure and punctual movements localized on patients' most painful areas along the masseter and temporal muscles and temporomandibular joint. The spherical surface concentrates vibratory stimulation on a smaller surface, with the aim of resolving muscle contractures and reducing myalgia.

### 2.4. Sample Size Estimation and Randomization

Since there were no data available from other clinical studies about the application of this kind of vibratory stimulation for TMD-related pain, patients were recruited using convenience sampling.

All local vibration devices (active and inactive) were randomly assigned to the study population using a random number generator (Research Randomizer©).

We used a total of 10 devices received from the manufacturer, 5 active and 5 inactive. These devices were delivered to patients by a single operator (G.S.); 34 active and 27 inactive devices were assigned over the course of the study. The devices showed the same exterior and functional characteristics. Neither the patients nor the operator knew which devices were active.

### 2.5. Statistical Analysis

Data analysis was performed with SPSS (version 23) statistical processing software. To assess whether there were significant differences in the pain levels (joint pain, muscular pain, and headache) of AG and IG patients at T0 and T1, a paired samples *t*-test was performed (statistical significance for *P* < 0.05).

## 3. Results

From the expected sample of 61 suitable patients, 7 were excluded for not carrying out the therapy according to the planned treatment modalities (Figure 1).

The resulting study sample therefore consisted of 54 patients; the characteristics of all study subjects are shown in Table 1.

**Figure 4:**
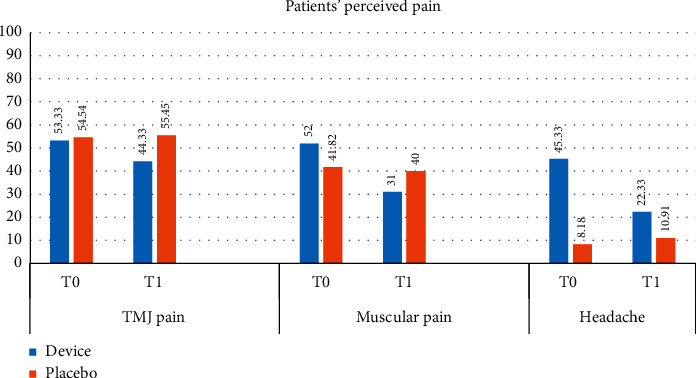
Average values of perceived pain in AG and IG at T0 and T1, according to VAS.

We found no significant differences comparing male and female subjects. Results for the group that carried out the therapy with active devices (AG) show a decrease between T0 and T1 in average values of all types of pain considered, with a statistically significant difference for TMJ pain, muscle pain, and headache. Results for the group that performed the therapy with inactive devices (IG) show a decrease in average values of muscular pain and an increase in the average values of TMJ pain and headache. In comparing data between the start (T0) and end of therapy (T1), Student's *t*-test was not significant for TMJ pain and muscular pain (Table 2 and Figure 4).

### 3.1. Device-Placebo Comparison

The Student's *t*-test analysis of the decrease in relative average pain values between patients who performed active and inactive therapy at T1 did not show significant results for TMJ pain, muscular pain, or headache (*P* > 0.05).

### 3.2. Treatment Effectiveness (PGI-I)

**Figure 5:**
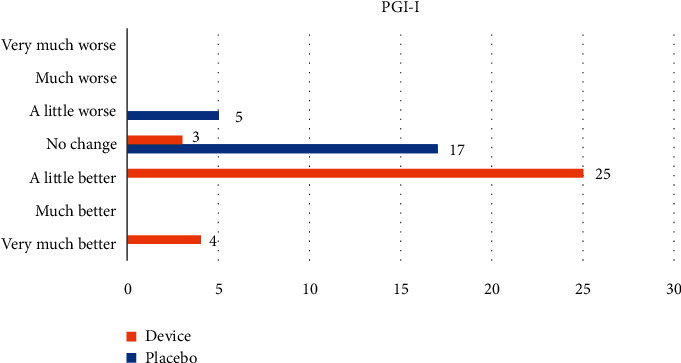
Patients' impression of the effectiveness of treatment according to the PGI-I Scale.

The results of patients' self-evaluations of treatment effectiveness using the PGI-I Scale are shown in Figure 5.

## 4. Discussion

This is the first study involving application of a local vibration device directly at the level of the joint area and masticatory muscles (masseter and temporalis) in order to evaluate the effectiveness of local vibration therapy for reducing TMD-related joint/muscular pain and headache.

The subjects of the group who underwent active local vibration therapy (AG) reported a significant decrease in average values of TMJ pain, muscular pain, and headache. Furthermore, there were no significant decreases in average pain values for patients in the study group that received placebo therapy with inactive devices (IG); these patients reported an increase in TMJ pain and headache that was statistically significant for the latter with respect to the initial pain level.

The choice to use vibratory stimulation in dysfunctional patients was based on evidence from previous studies showing the effectiveness of local vibration therapy in reducing chronic musculoskeletal pain and in delaying the onset of muscular pain [10, 11]. Several studies have shown that vibratory stimulus is capable of exciting afferents in both the Pacinian corpuscles and in the receptors of the skin, periodontium, muscle spindles, and tendon organs [15–17]. Moreover, from the gate control theory, we know that these sensory afferents can interact with the pain transmission pathways at the spinal level, causing modulation in response to the pain sensation [15, 18, 19]. All these mechanisms may contribute to the symptoms decrease observed in dysfunctional patients undergoing vibratory therapy in this study.

The pain symptomatology afflicting temporomandibular disorder patients is very complex and often invalidating, and it demonstrates a tendency to become chronic when there is no timely therapeutic intervention. There are several therapeutic strategies for relieving TMD-related pain, but only two studies evaluated the possible application of vibratory stimulation, and both report results in line with those obtained by our research.

Roy et al. [12] investigated the effect of vibrotactile therapy on resolution of chronic temporomandibular pain for a sample of 17 patients through the use of a stimulator that emitted vibrations of 20 Hz and 100 Hz. The results show the validity of this therapy in relieving TMD-associated pain, with a greater effectiveness at 100 Hz than 20 Hz for reducing muscular pain. Hara et al. [13] examined the analgesic efficacy of vibratory stimulation of an occlusal splint for a sample of 10 patients. The results highlighted significant variations in pain values on the VAS scale and on palpation, indicating the efficacy of the device in resolving TMD-related muscular pain.

In light of this evidence, the type of the device we used is particularly versatile, since it allows for daily home use for short periods and the possibility of extraoral application near the location of the pain. Patients who underwent therapy with an active device mostly reported an improvement in their pain condition and had no notable difficulty in following the home prescription. The extraoral application of the therapy also presents the additional advantage of being able to be applied in concomitance with the conventional therapy of occlusal splints, for patients needing mechanical support. Our study results reinforce the evidence that local vibration therapy is most effective for muscular and tension pain, such as local myalgia and headache. Our results regarding decrease in TMJ pain, however, also suggest that this therapy is able to resolve strictly articular problems. From this perspective, local vibration therapy could be a valuable addition to complement other conservative therapies.

This study also presents several limitations. First, despite the positive results obtained, the patient sample examined is still too limited to represent reliable and significant results regarding the efficacy of NOVAFON Pro Sk2/2 in reducing TMD-related symptoms. We see evidence of this limitation in the statistical nonsignificance, despite the encouraging clinical decrease of symptoms, of the compared average pain values between AG and IG at the end of therapy (T1) (with significance threshold set at 5%). Having noticed values close to the aforementioned significance threshold and in light of the limited sample size, the same test was carried out with an increased significance threshold of 10%. The results obtained from the second test show a significant difference regarding TMJ pain and headache with a *P* value of 0.08 and 0.06, respectively. To address this limitation and obtain more reliable results, the study sample is currently being expanded.

Second, the results obtained correspond to a single week of therapy, while prolonged evaluation, extending beyond the completion of therapy (follow-up), is necessary. Finally, pain assessment in this study was limited to patient self-assessment, but the importance of using multiple methods of pain assessment, given the complexity of changes this symptom can undergo during experimental procedures, has been well documented [20, 21].

## 5. Conclusion

Local vibration therapy is a valid support tool in the control of TMD-related familiar muscular pain. The extraoral application method is versatile, easy to apply, and integrates well with other conservative therapies; it is also useful for increasing patient compliance with other rehabilitation treatments. Moreover, this therapy offers the advantage of being performed at home by the patient, in different therapeutic moments, allowing the clinician greater possibility for treatment individualization.

Further studies are needed, however, to confirm the results obtained with larger samples and to include the short/long-term follow-up.

## Figures and Tables

**Table 1:** Characteristics of the study population.

Variable	Study group (AG), *N* = 32	Placebo group (IG), *N* = 22	Total, *N* = 54
Gender, no. (%)			
Female	24 (75.0)	19 (86.4)	43 (79.6)
Male	8 (25.0)	3 (13.6)	11 (20.4)
Age, mean (SD)	39.8 (9.9)	41.1 (9.9)	40.7 (9.9)

**Table 2:** Average values of perceived pain in AG and IG.

Study group-AG mean (SD)				Placebo group-IG mean (SD)		
Pain (VAS)	T0	T1	*P* value	T0	T1	*P* value
TMJ	53.33 (6.17)	44.33 (7.37)	0.0053^*∗*^	54.54 (21.15)	55.45 (20.18)	(NS)
Muscular	52.00 (26.70)	31.00 (21.75)	7.0223*E*−06^*∗∗*^	41.82 (22.28)	40.00 (19.49)	(NS)
Headache	45.33 (29.88)	22.33 (24.31)	1.3521*E*−05^*∗∗*^	8.18 (14.01)	10.91 (18.68)	0.0407^*∗*^

^*∗*^ *P* < 0.05 and ^*∗∗*^*P* < 0.001 in the difference T0–T1.

## Data Availability

The data used to support the findings of this study are available from the corresponding author upon request.
